# Experimental study of different oxides in B_2_O_3_–ZnO–BaO glass system for gamma-ray shielding

**DOI:** 10.1038/s41598-025-85230-9

**Published:** 2025-01-21

**Authors:** Mohamed Elsafi, M. I. Sayyed, Taha A. Hanafy

**Affiliations:** 1 Physics Department, Faculty of Science, Alexandria University, Alexandria, 21511 Egypt; 2 Department of Physics, Faculty of Science, Isra University, Amman, Jordan; 3 Department of Physics, Faculty of Science, University of Tabuk, Tabuk, Saudi Arabia; 4 Renewable Energy and Environmental Technology Center, University of Tabuk, Tabuk, 47913 Saudi Arabia

**Keywords:** Gamma-ray, Borate based glasses, Shielding, Attenuation coefficient, Transmission factor, Physics, Nuclear physics

## Abstract

Glass system of 45B_2_O_3_–20ZnO–30BaO–5X, (where X represents CaO, MgO, Al_2_O_3_, TiO_2_, CuO and Fe_2_O_3_) in mole percentage was investigated for gamma ray radiation shielding experimentally. Six glass composites were fabricated and the density was measured experimentally and the BZBCa glass sample has the least density with a value of 3.932 g cm^−3^ and this is due to the presence of CaO in it, and the sample BZBFe has the highest density with a value of 4.031 g cm^−3^. Through comparing the linear attenuation coefficient (LAC) data (experimental and Phy-X) for the BZBX glass samples, the LAC values for glass samples obtained experimentally and using Phy-X are in a very close range. All the glass samples have the greatest LAC values at 0.0595 MeV, the lowest energy value. Sample BZBCu has a LAC value of 16.203 1/cm, which is also the highest LAC value among all the studied glasses, this is as a result of the high density of this glass and due to the high atomic number of Cu. The glasses’ transmission factor (TF) at 1 cm thickness against energy was determined. The TF values of all the glasses were almost zero. The TF values increased significantly for all the glasses when the energy was increased to 0.662 MeV, and for sample BZBCa its TF value increases 74.08%, which was the highest TF value increase. The half-value layer and other shielding parameters have been determined experimentally.

## Introduction

Our environment is not devoid of radiation, whether the radiation is natural or man-made. Natural radiation is cosmic rays coming from space and radiation coming from the interior of the Earth, whether from radioactive rocks, global warming, etc. Meanwhile, industrial radiation is employed in many fields including agriculture, industry, and medicine. In the medical field, radiation is used in surgery, treatment, and radiological diagnosis, and in the agricultural field, radiation is used to fertilize soil, nourish plants, preserve agricultural food products, and develop animal production. In the industrial field, radiation is used in industrial radiography, industrial metrology, well logging, calibration systems, etc. As radiation spreads, protection must be provided because it is harmful to living organisms and can cause tissue damage and cancer if a person has exposure to a large radiation dose. Therefore, protection from radiation reduces the health risks resulting from it^1–5^.

One of the main radiation protection factors is the manufacture of shielding materials. There are many materials that are used to protect against ionizing radiation^5^. Concrete is used for its low cost and high durability, but it can degrade over time and is an inelastic material^6^. Lead is an effective material against ionizing photons due to its high atomic number and density, but it is considered a toxic substance^7^. Boron is considered a highly efficient neutron absorber, but it does not show its efficiency with photons and other particles^8^. Because of these limitations, there has been and continues to be continuous development in preparing shielding materials that provide the least environmental damage, the highest shielding efficiency, and are suitable for the place to be protected.

Density is known to play an important role in the attenuation process. Recently, glass systems of different densities have been manufactured, and each glass compound has different properties. We are always looking for materials that are environmentally friendly, low in cost, adaptable and durable, in addition to good protection against ionizing photons and neutrons^9,10^. These properties are available in glass, and it is possible to change the density of the compound easily by replacing some oxides with others. In addition to this, it is possible to obtain a transparent glass composition that has all the mentioned properties. For this reason, glass is suitable as a shielding material in industrial and medicine applications^11,12^.

There has been the recent use of borate glasses in myriad applications because they are characterized by good durability, mechanical, and thermal properties, and also the addition of heavy metals gives them a high ability to attenuate radiation^13–21^. Adding barium oxide and zinc oxide to borate glasses gives the compound an increase in density in addition to improving thermal stability and improving its mechanical properties. In this work, the proportions of B_2_O_3_, BaO and ZnO in the compound were fixed, and different types of other oxides were added, such as titanium oxide, iron oxide, and copper oxide. The addition of these oxides gives different properties in terms of transparency and density, in addition to thermal and attenuation properties^22–25^.

The use of the experimental method in shielding measurements is important before using the shielding material in various applications, because it gives realistic results for the shielding composite. Experimental measurement requires the presence of radioactive point gamma source (emits single or multi-lines) and a detector works to determine the radiation falling on it and calculate its intensity, as well as a lead collimator so that a narrow beam can be obtained during the measurement. Before measuring, the detector should be calibrated in terms of energy and efficiency, and the distances between the source, the shielding material, and the detector should be adjusted^26–29^.

In this work, an experimental technique was utilized to determine the radiation attenuation performance of 45B_2_O_3_–20ZnO–30BaO–5 (Metal Oxide), since the metal oxides used were TiO_2_, Al_2_O_3_, CaO, CuO, Fe_2_O_3_ and MgO. Different gamma point sources such as Co-60, Am-241 and Cs-137, in addition a HPGe-detector (semiconductor high purity germanium detector) were used in the measurements. The linear attenuation coefficient (LAC) and the radiation shielding efficiency (RSE), as well as other shielding parameters were determined and compared with other commercial materials for shielding purposes.

## Materials and methods

### Fabrication of the glass system

**Fig. 1:**
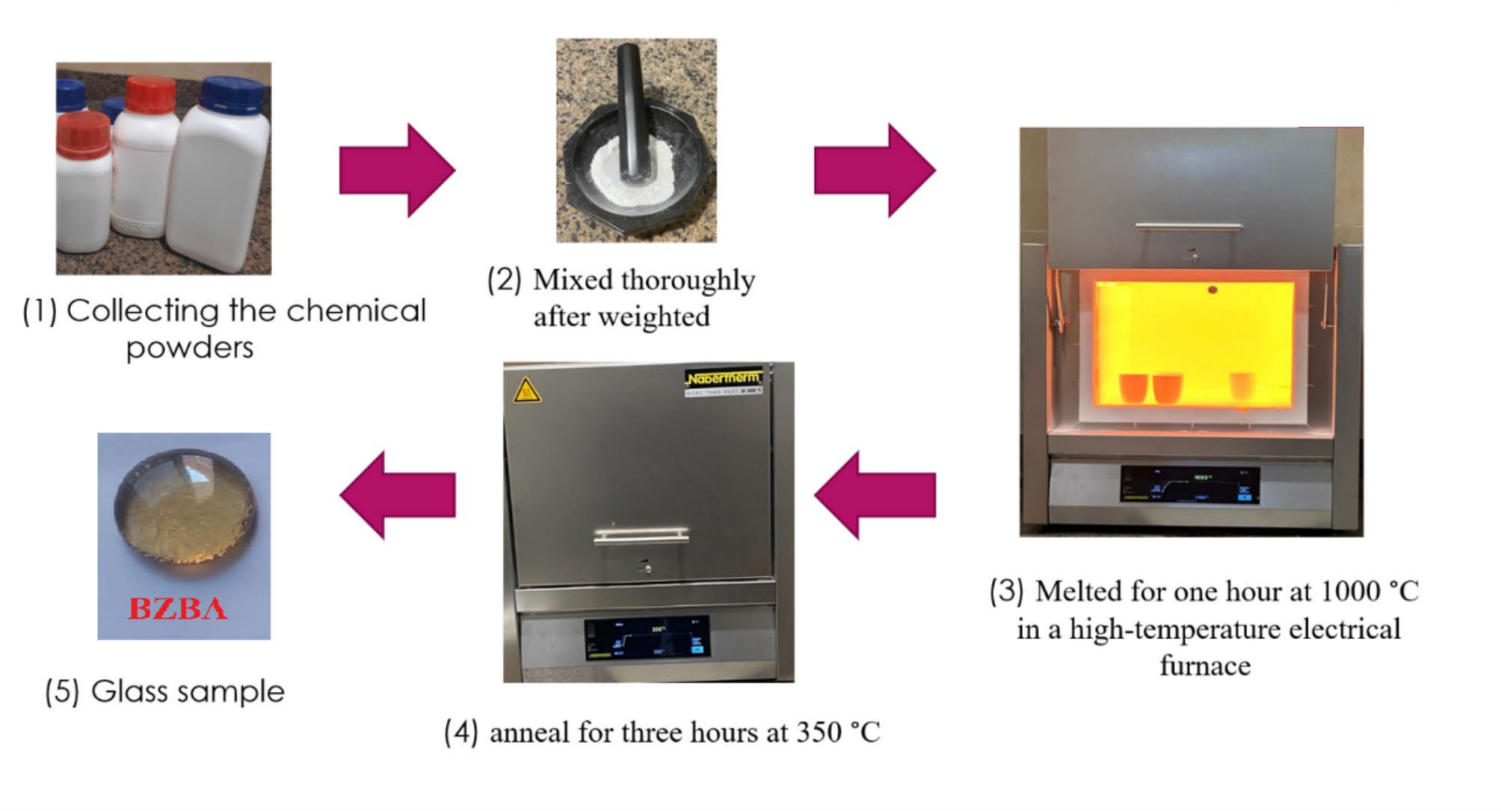
Steps of glass preparation.

**Fig. 2:**
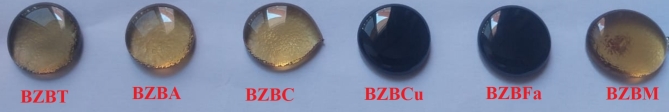
The real image of prepared glasses.

Six glass composites were prepared according to the glass system (45B_2_O_3_–20ZnO–30BaO–5X), (where X represents CaO, MgO, Al_2_O_3_, CuO, TiO_2_ and Fe_2_O_3_) in mole percentage via the melt-quenching method. The powders B_2_O_3_, ZnO, BaO and X-oxide were purchased from Sigma Aldrich Chemicals with ~ 99.8% purity. The powders were mixed, putted in aluminum crucible, heated to 1000 °C in electric furnace, the mixed powder was melted, transferred to another furnace with 350 °C for annealing, left inside the furnace till reach the room temperature and the glass was formed. The preparation steps can be summarized in Fig. 1 and the real picture of prepared glass is shown in Fig. 2. Table 1 tabulates the present glasses’ chemical composition.

**Table 1:** The chemical composition and densities of prepared glasses.

Glass name	Compositions (mol, %)									Density (g/cm^3^)
	B_2_O_3_	ZnO	BaO	TiO_2_	Al_2_O_3_	CaO	CuO	Fe_2_O_3_	MgO	
BZBT	45	20	30	5	0	0	0	0	0	3.965 ± 0.006
BZBA	45	20	30	0	5	0	0	0	0	3.955 ± 0.007
BZBCa	45	20	30	10	0	5	0	0	0	3.932 ± 0.009
BZBCu	45	20	30	10	0	0	5	0	0	4.014 ± 0.010
BZBF	45	20	30	10	0	0	0	5	0	4.031 ± 0.008
BZBM	45	20	30	10	0	0	0	0	5	3.944 ± 0.011

### Physical properties

The density of BZBX-glass sample is determined using the Archimedes method, which is based on the principle of buoyancy as shown in Eq. (1)^22^.

1 $$\rho = \frac{{m_{r} }}{{m_{r} - m_{L} }}$$

where m_r_ and m_L_ represents the mass in both liquid and air, respectively, in the case of water being used as the immersing liquid. By knowing the total molecular mass of the glass (*M*), the molar volume can be calculated by next equation^22^:

2 $$V_{m} = \frac{M}{\rho }$$

The packing density (V_t_) and oxygen molar volume (V_o_) have been deduced from the molar volume as given in the formula below:

3 $$V_{t} = \mathop \sum \limits_{i} \frac{{V_{i} x_{i} }}{{V_{m} }}$$

4 $$V_{o} = \mathop \sum \limits_{i} \frac{{V_{m} }}{{n_{i} x_{i} }}$$

where V_i_ represent the packing factor of the oxide calculated from its ionic radii^30,31^, ${x}_{i}$ and ${n}_{i}$ are the mole fraction and the fraction of oxygen atoms for each component oxide. The Poisson’s ratio (σ) can be obtained from packing density by using the following equation:

5 $$\sigma = 0.5 - \frac{1}{{7.2V_{t} }}$$

The refractive index of the prepared samples can be determined by next relation^32,33^:

6 $$n = \frac{\rho + 10.4}{{8.6}}$$

From the refractive index, the dielectric constant ($\varepsilon$) and the reflection loss (R) can be calculated by the following formula^34,35^:

7 $$\varepsilon = n^{2}$$

8 $$R = \left( {\frac{n - 1}{{n + 1}}} \right)^{2}$$

### Attenuation parameters measurements

**Fig. 3:**
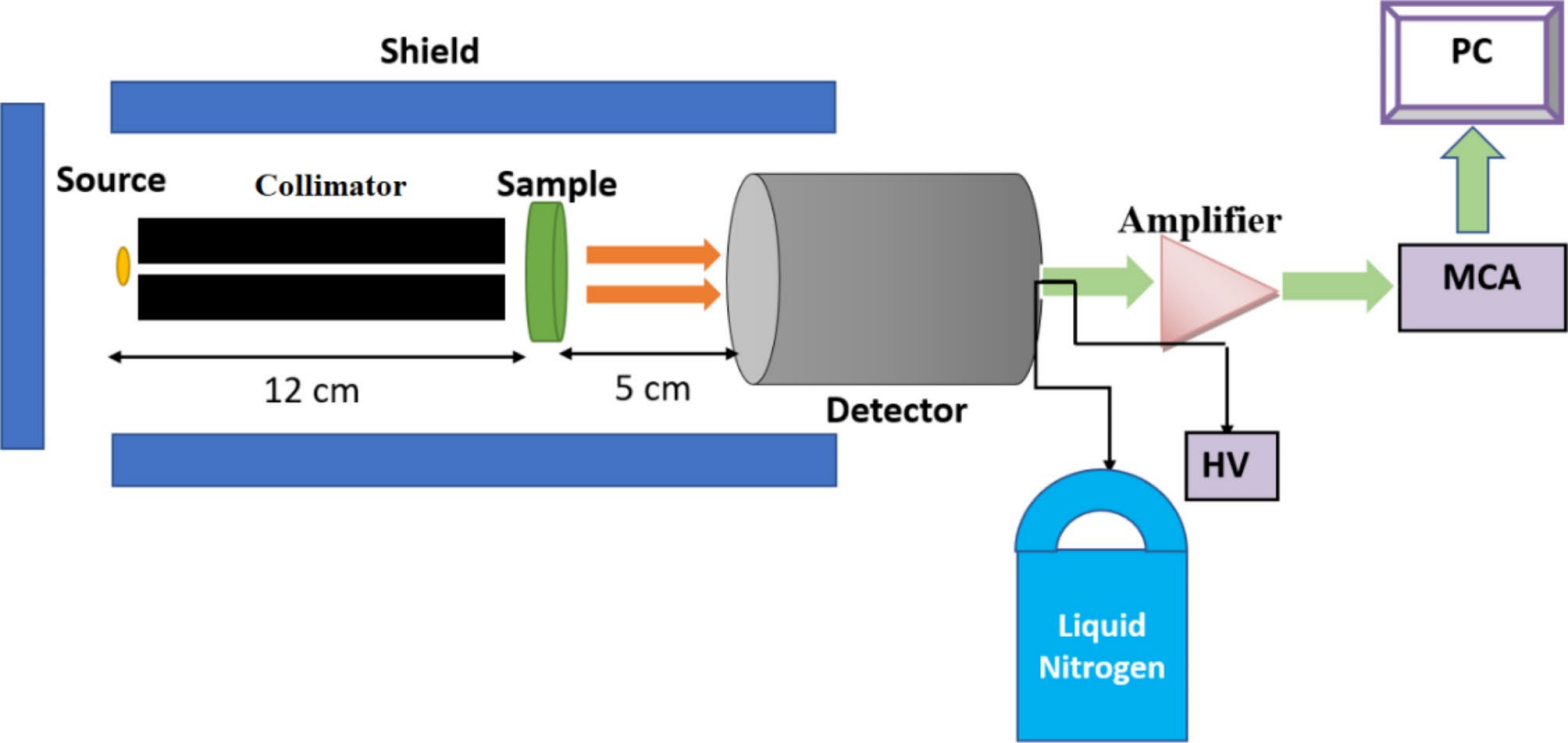
Setup of experimental work.

**Fig. 4:**
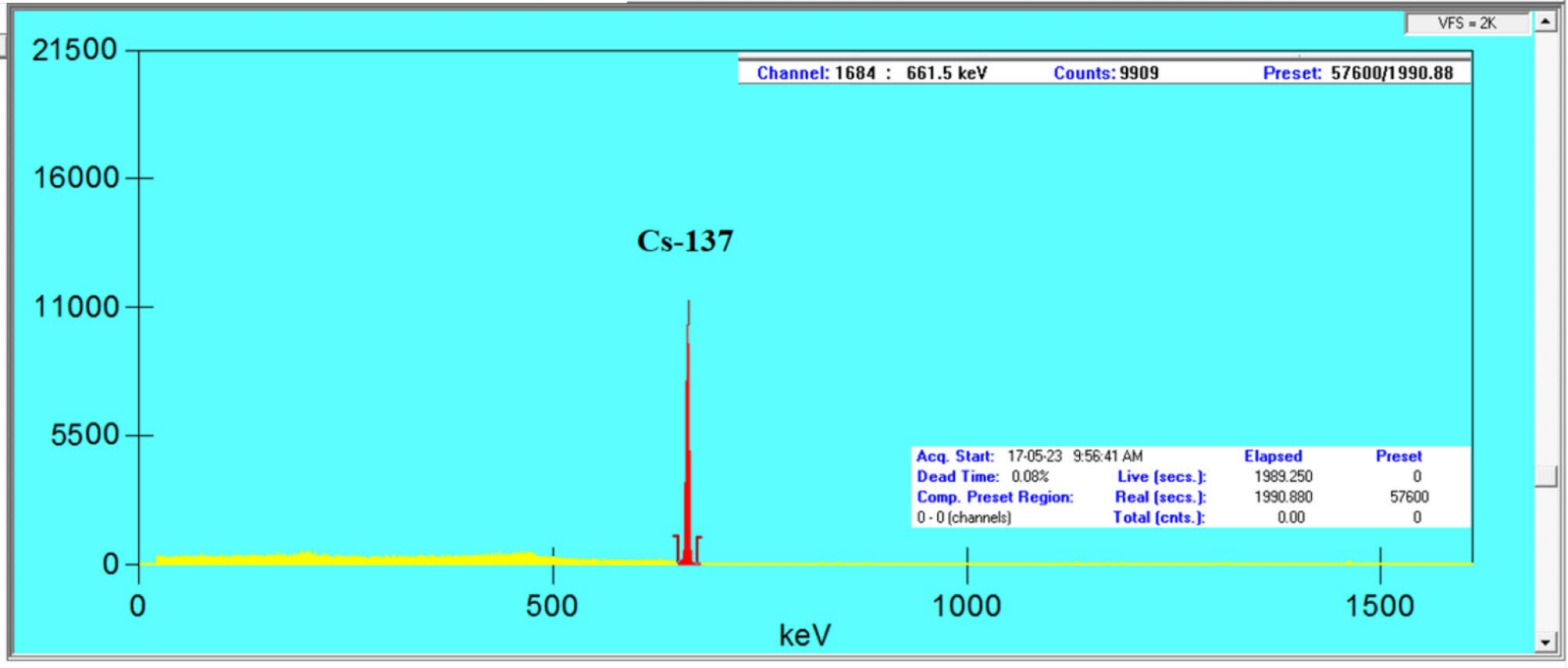
Gamma ray spectrum for Cs-137 point source in case BZBT glass sample.

For the investigated glasses, Monte Carlo simulations were previously used to study their radiation shielding properties at specific energies^3,4^, while in this study, experimental methods were employed to measure these properties. This experimental approach provides direct measurements and enhances the understanding of the glasses’ shielding performance. Experimental work offers tangible results that reflect real-world conditions, highlighting differences that may not be captured through simulations. The current glass samples’ gamma-ray shielding parameters were experimentally measured via the narrow beam method, as shown in Fig. 3. The components include a gamma point sources with initial activity 1.48 kBq at 1 September 1998 (Am-241, Cs-137, and Co-60), a lead collimator (inner hole diameter: 8 mm), and a HPGe or high purity germanium detector with relative efficiency 24% and energy resolution 1.96 keV at second line of Co-60 (1.333 keV). The energy range of the point source emission is 0.060 to 1.333 MeV. The energy and efficiency calibration were done for the detector using this point sources and before the measurement takes place, calibration of the detector is required using a material with known attenuation, as is the glass sample’s position between the detector and the gamma source. Under identical conditions, the detector is run once with the sample and once without the glass sample (with thickness ranges from 3.4 to 4 mm) to determine the net count rate or the area under the photopeak for both cases, which are coded as C and C_o_, respectively. The detector is operated for a sufficient time to obtain the lowest error in the area measurements (< 1%) or to obtain 10,000 counts. The time here varies from one source to another. Cs-137 and Am-241 do not complete 20 min, while Co-60 takes an hour to obtain an error of less than 1% due to its half-life. From the net count rate which calculated using Genie-2000 software as shown in Fig. 4 and the measured glass sample’s thickness (t), it is possible to calculate the *LAC*, which is defined by the likelihood of gamma interaction through the glass sample’s linear thickness, and can be determined using Eq. (1)^36–40^:

9 $$LAC = \frac{1}{t}\ln \frac{{C_{0} }}{C }$$

Other important shielding factors, such as half-value layer (HVL) and tenth-value layer TVL), mean free path (MFP) and radiation protection efficiency (RPE), measure the ability of shielding materials to resist incident radiation, and can be found through Eqs. (2)–(5), which can also express and calculate N and N_0_^41,42^:

10 $$HVL = \frac{Ln \left( 2 \right)}{{LAC}}$$

11 $$MFP = \frac{1}{LAC}$$

12 $$TVL = \frac{{Ln \left( {10} \right)}}{LAC}$$

13 $$RPE, \% = \left[ {1 - \frac{{C_{0} }}{C }} \right] \times 100$$

## Results and discussion

**Fig. 5:**
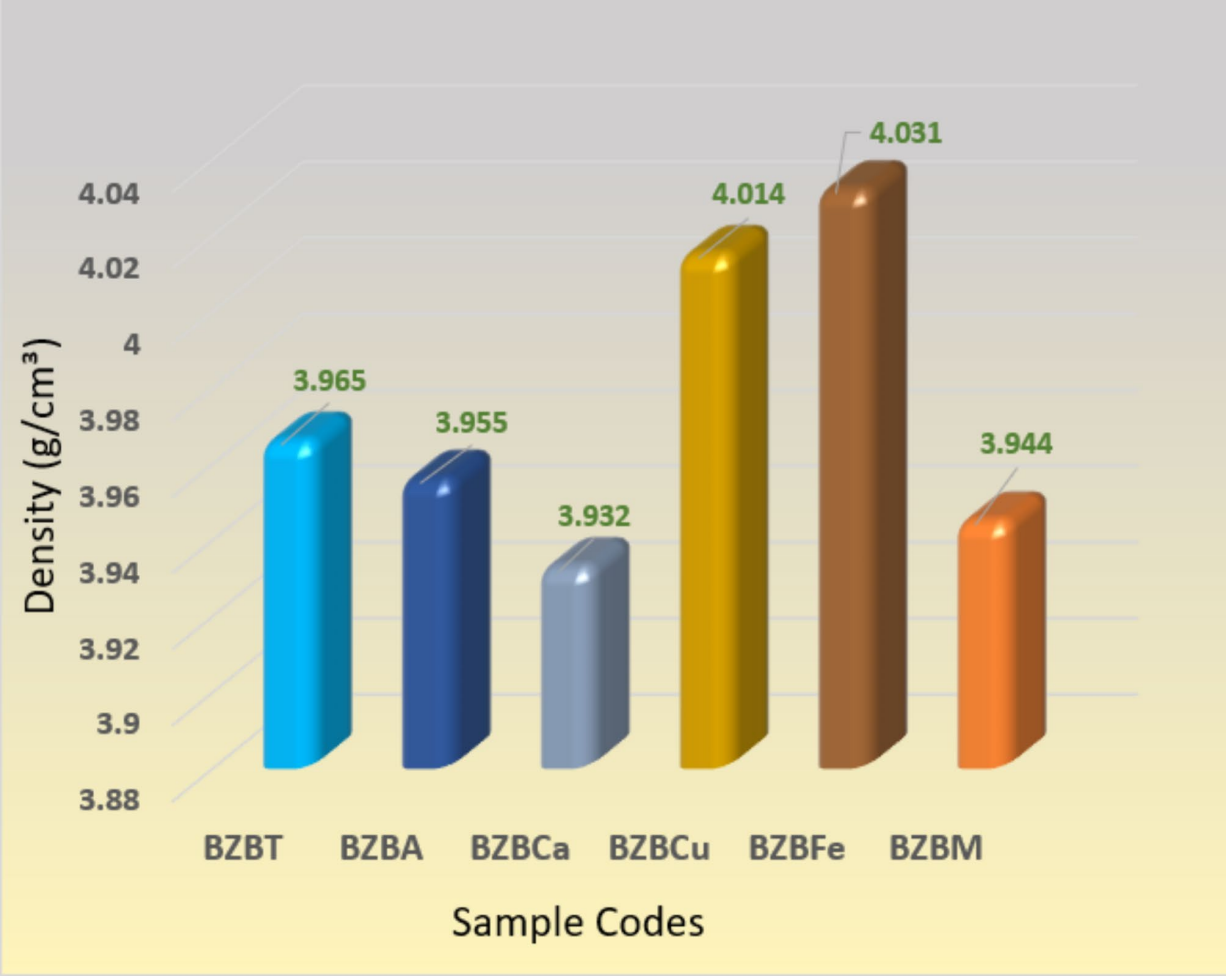
A graph of density for the studied glasses.

Figure 5 plots the fabricated glass sample’s density. Clearly, BZBCa has the least density with a value of 3.932 g cm^−3^ and this is due to the presence of CaO in it, followed by sample BZBM (3.944 g cm^−3^), sample BZBA (3.955 g cm^−3^), sample BZBT (3.965 g cm^−3^), sample BZBCu (g cm^−3^), while sample BZBFe has the highest density of 4.031 g cm^−3^ as a result of the presence of Fe_2_O_3_ in it which increases it’s atomic weight. Table 2 shows some physical properties of the fabricated glass such as molar volume (V_m_), packing factor (V_t_), oxygen molar volume (V_o_) and refractive index, where the results showed that most of the properties are close to each other for the different prepared glass systems.

**Table 2:** Physical properties of fabricated glasses.

Sample codes	V_m_	V_t_	V_o_	Poisson ratio	Refractive index	Dielectric constant	Reflection loss
BZBT	24.61	0.634	12.623	0.281	1.670	2.790	0.063
BZBA	24.96	0.639	12.478	0.283	1.669	2.786	0.063
BZBCa	24.52	0.626	12.905	0.278	1.667	2.777	0.062
BZBCu	24.31	0.628	12.795	0.279	1.676	2.809	0.064
BZBF	25.20	0.634	12.601	0.281	1.678	2.816	0.064
BZBM	24.24	0.629	12.760	0.279	1.668	2.782	0.063

**Fig. 6:**
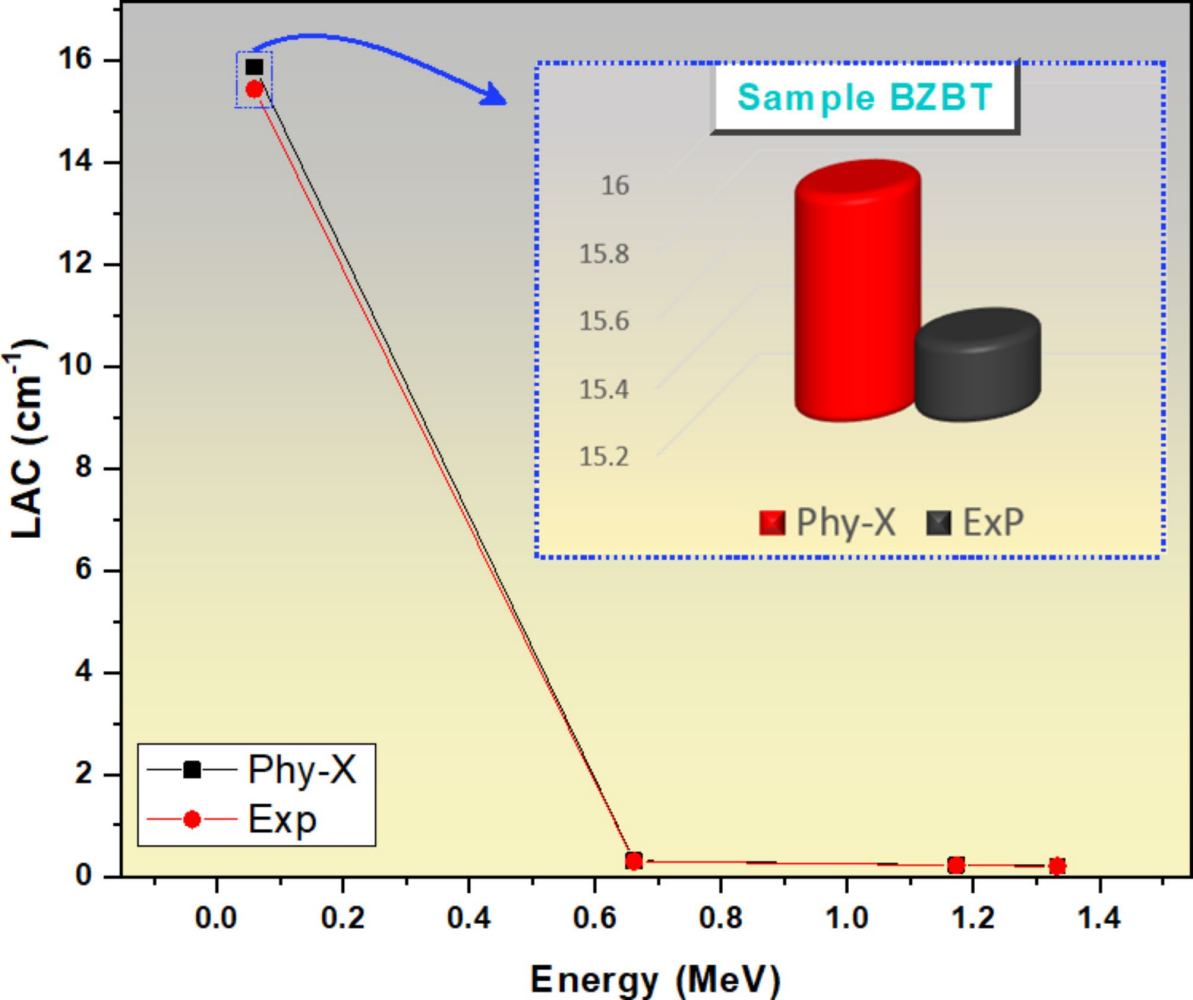
A comparison graph of the LAC value for glass sample BZBT obtained experimentally and the one obtained using Phy-X.

In terms of the BZBT glass sample, Fig. 6 compares the LAC data (experimental and Phy-X). As shown in Fig. 6 there’s a very close range in the LAC values for sample BZBT obtained experimentally as well as the one obtained using Phy-X. For example, at the lowest energy with a value of 0.060 MeV, the two results obtained has their highest values; result obtained by Phy-X is the highest with a LAC value of 15.875 cm^−1^ while experimentally the value was 15.438 cm^−1^. With a 0.060 to 0.662 MeV energy increase, the LAC values dropped drastically as shown in Fig. 6, where the experimental value decreases to 0.292 cm^−1^, while the Phy-X value decreased to 0.302 cm^−1^. At 1.33 MeV, the experimental (0.198 cm^−1^) and Phy-X values (0.204 cm^−1^) are in good agreement. The experimental LAC with its uncertainty compared with theoretical values is tabulated in Table 3.

**Table 3:** The experimental LAC (cm^−1^) with its uncertainties and Phy-X values at different energies.

Energy (MeV)	BZBT			BZBA			BZBCa		
	Phy-X	Exp	Dev%	Phy-X	Exp	Dev%	Phy-X	Exp	Dev%
0.060	15.875	15.438 ± 0.043	2.83	15.620	15.045 ± 0.032	3.82	15.910	15.254 ± 0.037	4.30
0.662	0.302	0.292 ± 0.013	3.27	0.302	0.293 ± 0.021	2.76	0.300	0.290 ± 0.009	3.30
1.173	0.218	0.215 ± 0.010	1.28	0.218	0.211 ± 0.009	3.22	0.217	0.213 ± 0.018	1.87
1.333	0.204	0.198 ± 0.009	3.22	0.204	0.200 ± 0.011	1.88	0.202	0.198 ± 0.010	2.11

Energy (MeV)	BZBCu			BZBF			BZBM		
	Phy-X	Exp	Dev%	Phy-X	Exp	Dev%	Phy-X	Exp	Dev%
0.060	16.203	15.522 ± 0.039	4.39	15.710	15.255 ± 0.029	2.98	16.047	15.315 ± 0.027	4.78
0.662	0.306	0.296 ± 0.022	3.22	0.307	0.298 ± 0.018	2.95	0.301	0.291 ± 0.013	3.33
1.173	0.221	0.214 ± 0.012	2.98	0.222	0.218 ± 0.011	1.89	0.217	0.211 ± 0.008	2.88
1.333	0.206	0.200 ± 0.019	3.21	0.208	0.201 ± 0.008	3.22	0.203	0.197 ± 0.009	3.11

**Fig. 7:**
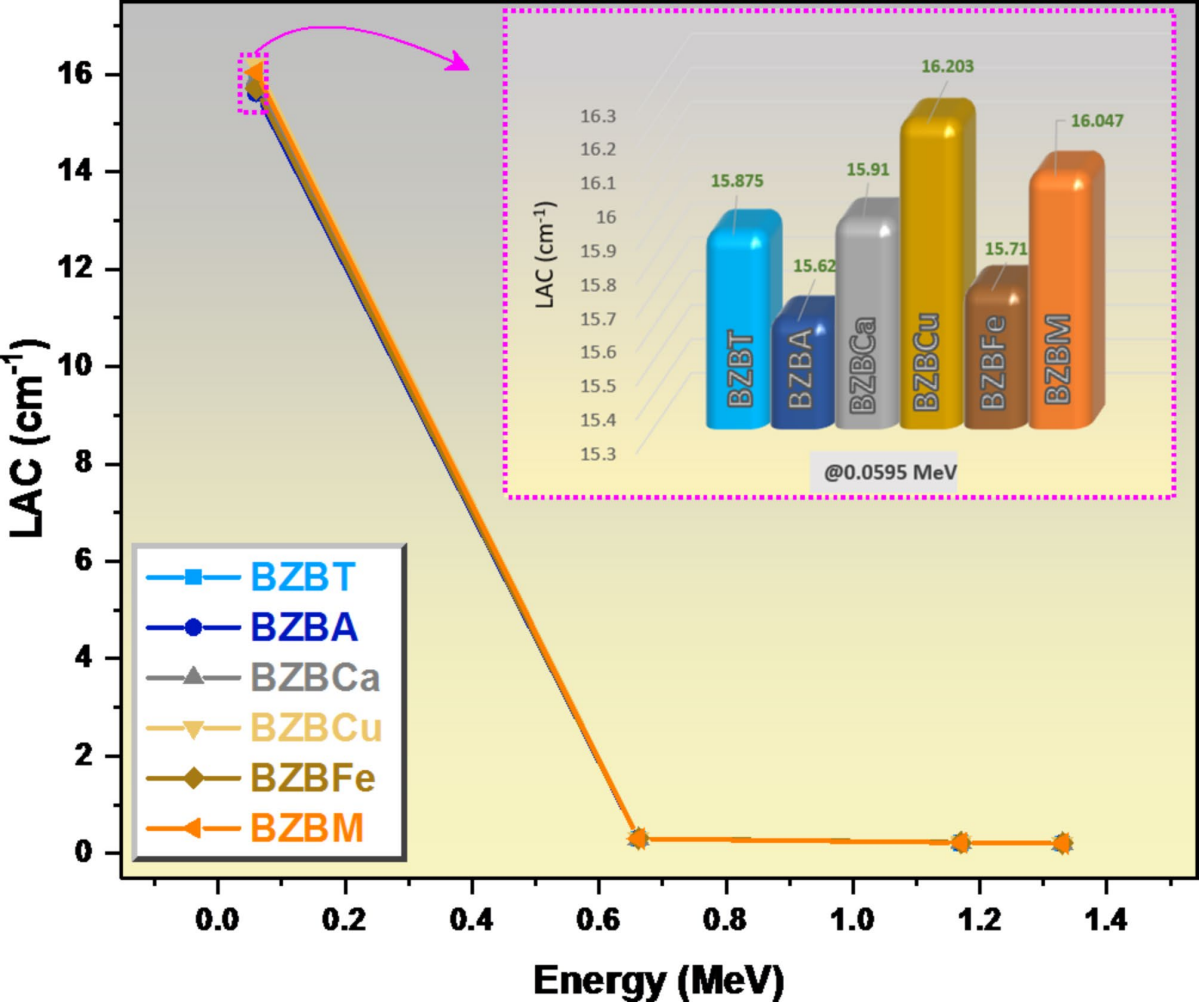
A graph of LAC verus energy for the studied glasses.

Figure 7 compares the LAC for the different compositions at the examined energy levels. As expected, all the glass samples have the greatest LAC values at 0.0595 MeV, the lowest energy value. Sample BZBCu has a LAC value of 16.203 /cm, which is also the highest LAC value among all the studied glasses, this is as a result of the high density of this glass and due to the high atomic number of Cu. BZBA glass sample has a relatively low LAC and this is due to the low density of Al_2_O_3_. With a 0.0595 to 0.662 MeV energy increase, as shown in Fig. 7, we observed a great decrease in all the glass samples’ LAC values, with sample BZBCu experiencing the greatest decrease in LAC value from 16.203 cm^−1^ to 0.306 cm^−1^, while sample BZBA experiences the least decrease in LAC from 15.620 cm^−1^ to 0.302 cm^−1^. With a further 0.662 to 1.33 MeV energy increase, the decrease in the LAC values became lower, with sample BZBCu decreasing from 0.306 cm^−1^ to 0.206 cm^−1^ which was the highest observable decreased in LAC value due to the presence of CuO. Also, sample BZBFe which is the second highest observable decreased in the LAC due to the presence of Fe_2_O_3_ (this glass’s LAC reduces from 0.307 cm^−1^ to 0.208 cm^−1^). Hence samples BZBFe and BZBCu are the better candidate as a radiation attenuation material.

**Fig. 8:**
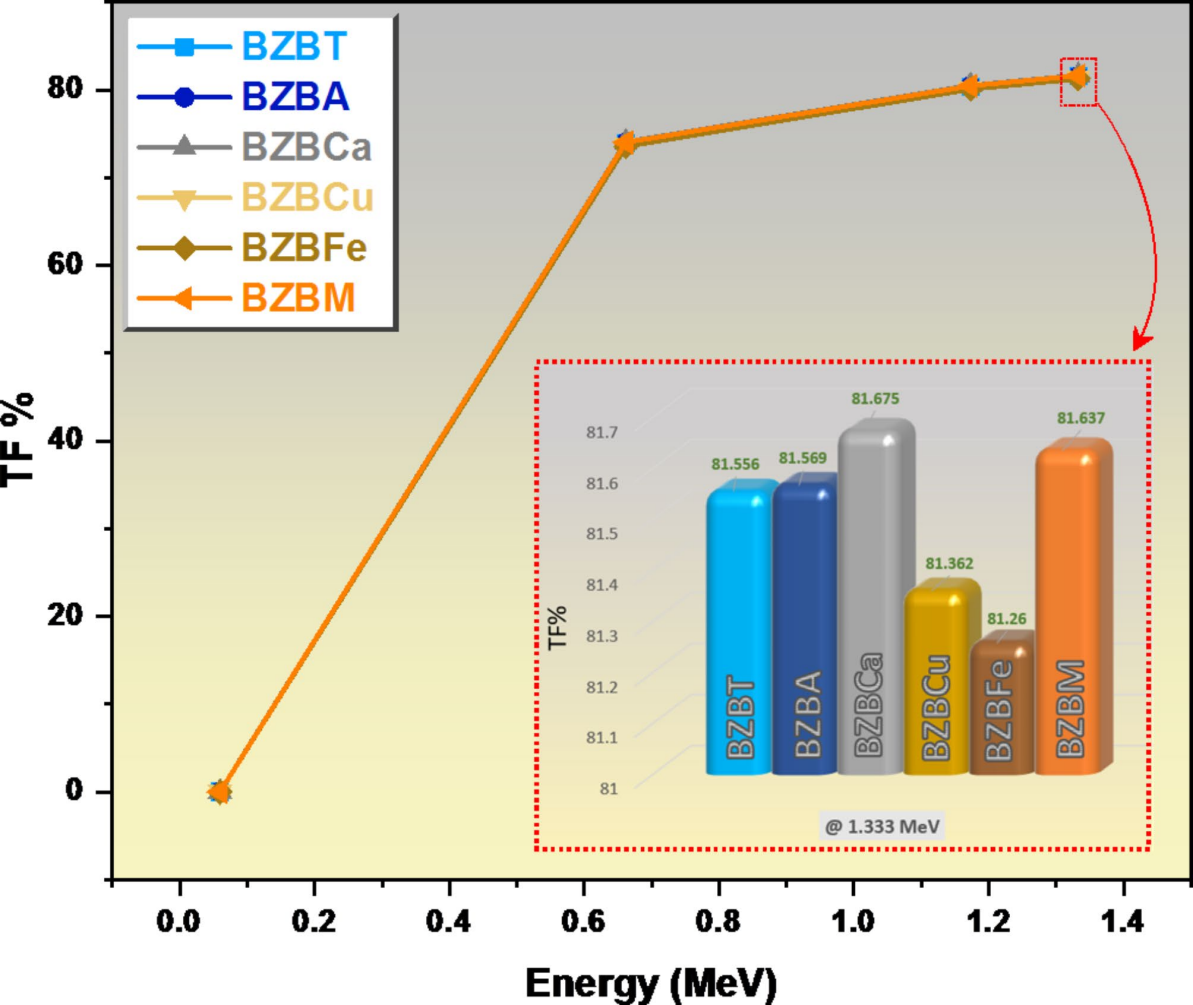
A chart of the transmission factor TF of all the glasses at 1 cm thickness against energy.

In Fig. 8, we plotted all the glasses’ transmission factor (TF) at 1 cm thickness against energy. When the incoming radiation was at very low energy (0.060 MeV), none of the glasses transmit the radiation and it was completely absorbed, as shown in Fig. 8, meaning the TF values of all the glasses were almost zero. Immediately as the radiation energy was increased to 0.662 MeV, the TF values increase significantly for all the glasses: for sample BZBCa its TF value increases 74.08%, which was the highest TF value increase due to the presence of Ca which has low atomic number; sample NZBM increases to 74.02%, sample BZBA increases to 73.97%, sample BZBT increases to 73.93%, followed by sample BZBCu which increases to 73.66%, and then the least increase was observed in sample BZBFe which increases to 73.57% due to the presence of Fe_2_O_3_. Further increase in the energy of the radiation from 0.662 MeV to 1.333 MeV shows a slow increase in the TF values for all the glasses and the TF for all glasses varied between 81.68% and 81.36%. The TF is very high at 1.333 MeV which means that most of the photons with high energy can penetrate these glasses.

**Fig. 9:**
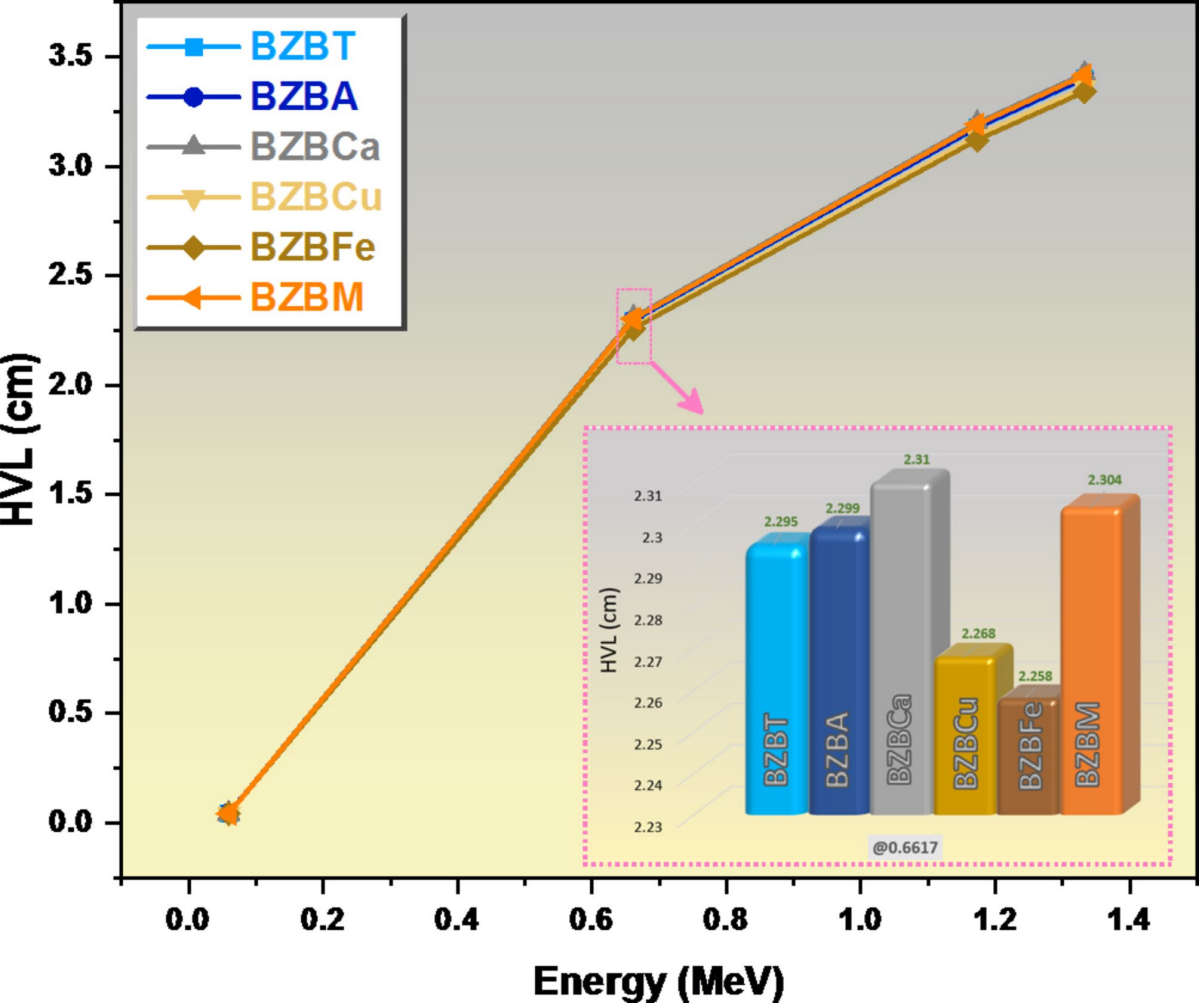
A graph of the half value layer HVL against energy for the glass samples.

Figure 9 shows the HVL for the prepared glasses. The HVL at a very low energy value of 0.060 MeV was very small across all the glass samples as seen in Fig. 9. At this energy, the HVL is almost 0.044 cm for the glasses and this indicated a very thin layer of these glasses can be successfully attenuating the low energy photons. We observed a sudden increase in the HVL values of all the glasses as the energy increases from 0.060 MeV to 0.662 MeV with sample BZBCa increasing to 2.310 cm, sample BZBM increases to 2.308 cm, sample BZBA increases to 2.299 cm, sample BZBT increases to 2.295 cm, sample BZBCu increases to 2.268 cm, while sample BZBFe increases to 2.258 cm which was observed to be the least increase. It is Cleary seen the difference in the thickness of the glass sample needs to attenuate the photons with low and high energy levels, a layer of 0.044 cm is required in the case of shielding low energy radiation, while we need a layer with thickness greater than 2 cm in case of shielding the photons with energy of 0.622 MeV. As the energy increases to 1.333 MeV, the thickness needed to attenuate the photons also increases and reached to around 3.5 cm. Within the range of the energy considered, sample BZBFe and BZBCu has the least increase in HVL which invariably makes these glasses good shielding material.

**Fig. 10:**
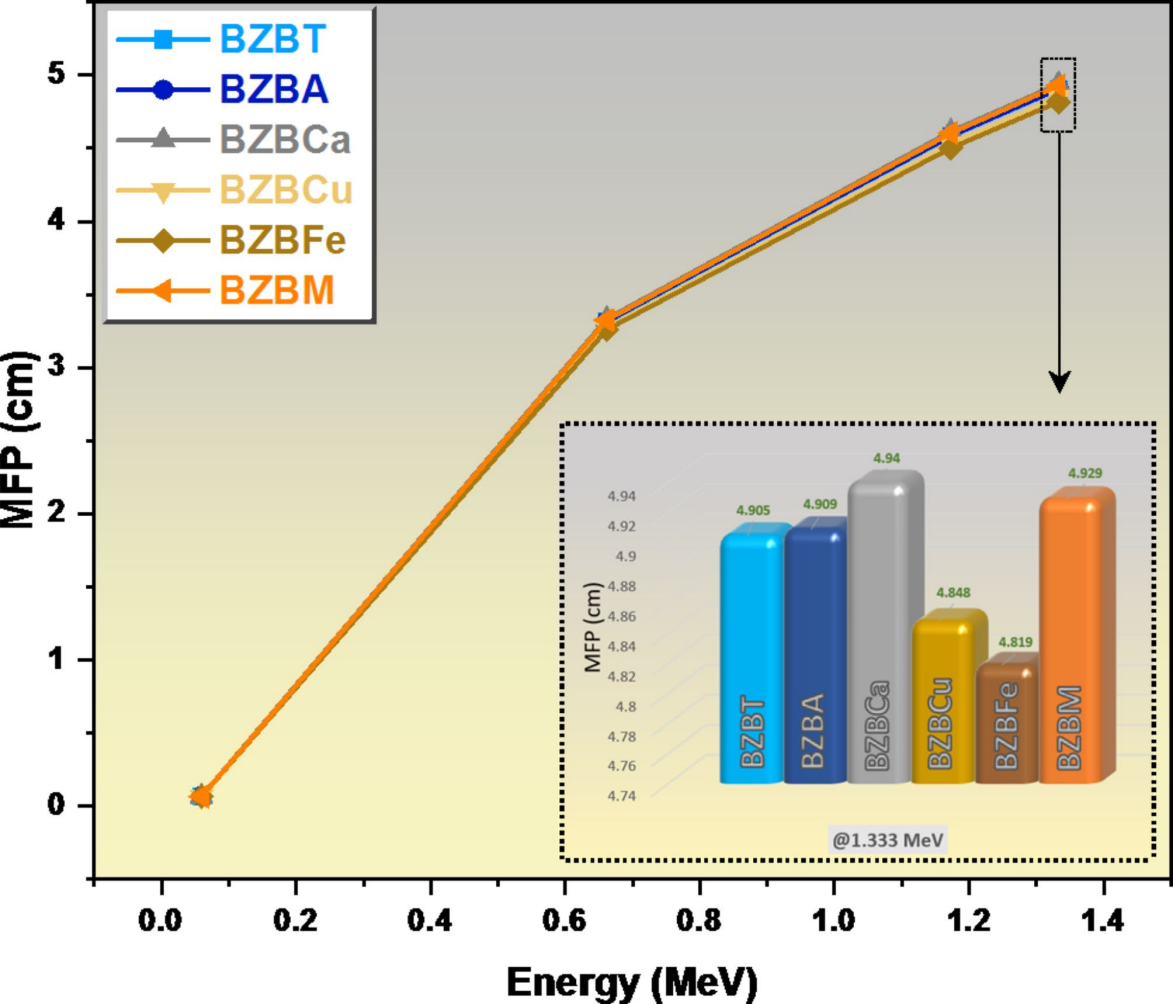
A graph of mean free path MFP against energy.

Based on Fig. 10, the MFP of the glass materials was very low at the lowest energy of the radiation (0.060 MeV) and this is the same trend reported to the HVL in the previous figure. At 0.06 MeV, the MFP is around 0.064 cm for the prepared glasses, increasing to around 3.258–3. 333 cm at 0.622 MeV. The MFP results have almost the same trend with the HVL and the difference only in the values. The MFP at 1.333 MeV is in order of 4.819 and 4.940 cm.

**Fig. 11:**
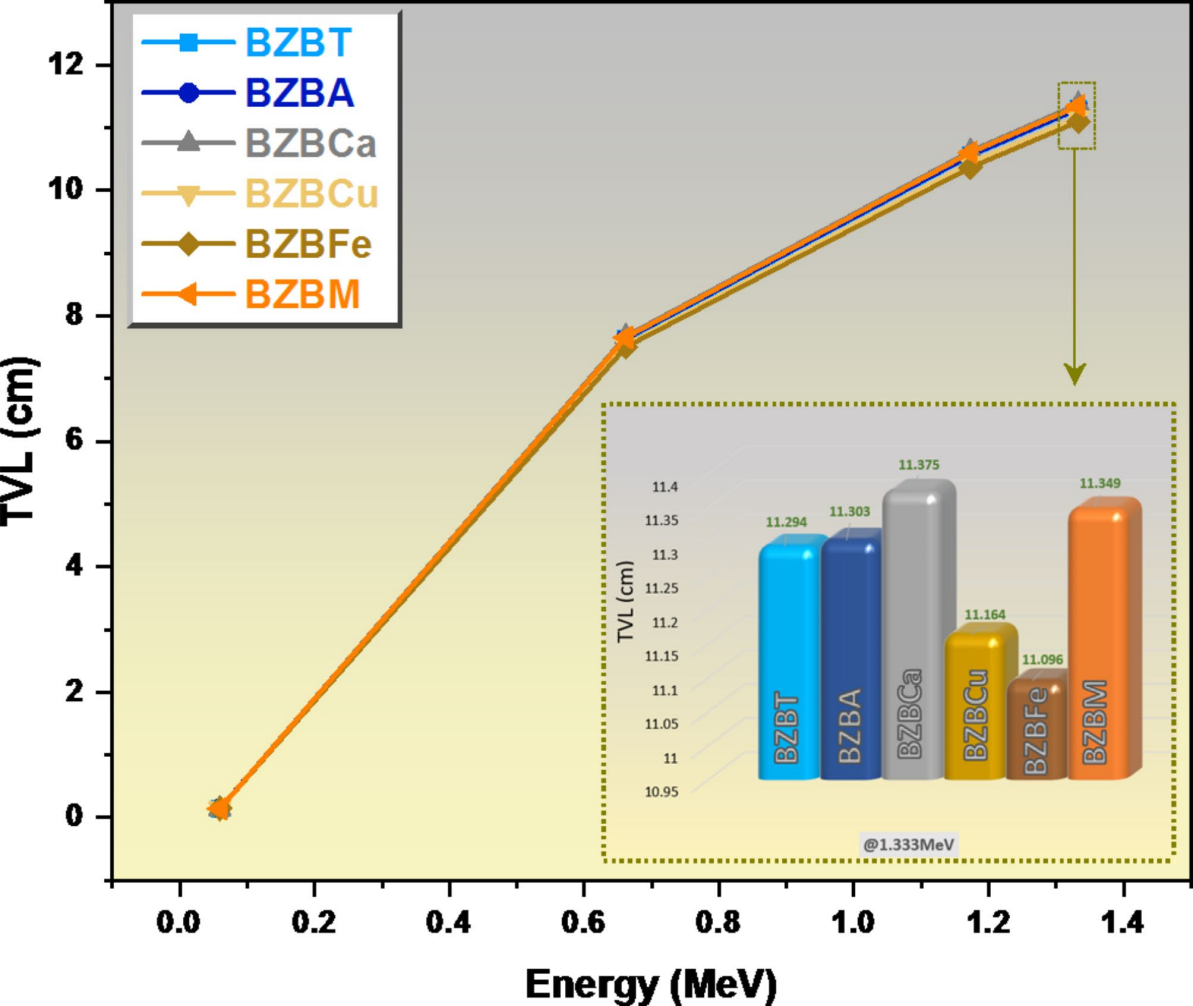
A chart of tenth value layer TVL of the glasses against energy E.

The required thickness of a glass sample that is capable of attenuating 90% of the incoming radiation was plotted in Fig. 11 against the energy of the radiation, and as expected, at a lower energy level of the radiation, 0.060 MeV, the required thickness is relatively low for all the glass samples. For example, sample BZBT and sample BZBCa have the same TVL value of 0.145 cm, sample BZBA and sample BZBFe also have the same TVL value of 0.147 cm, while sample BZBCu has a TVL value of 0.142 cm and lastly sample BZBM has a TVL value of 0.143 cm. We noticed a drastic increase in the TVL values across all the glass samples as we increased the energy from 0.060 to 0.662 MeV whereby sample BZBCa shows the highest increase in TVL value from 0.145 cm to 7.674 cm, sample BZBM increased from 0.143 to 7.654 cm, then sample BZBA which increased from 0.147 to 7.636 cm, sample BZBT increased from 0.145 to 7.623 cm, followed by sample BZBCu which increased from 0.142 to 7.532 cm and lastly sample BZBFe increased from 0.147 to 7.503 cm. The TVL values at 1.333 MeV are high and equal to 11.375 cm for BZBCa and 11.096 cm for BZBFe. As we found in the HVL, the TVL for BZBFe and BZBCu are lower than the TVL for the remaining glasses.

**Fig. 12:**
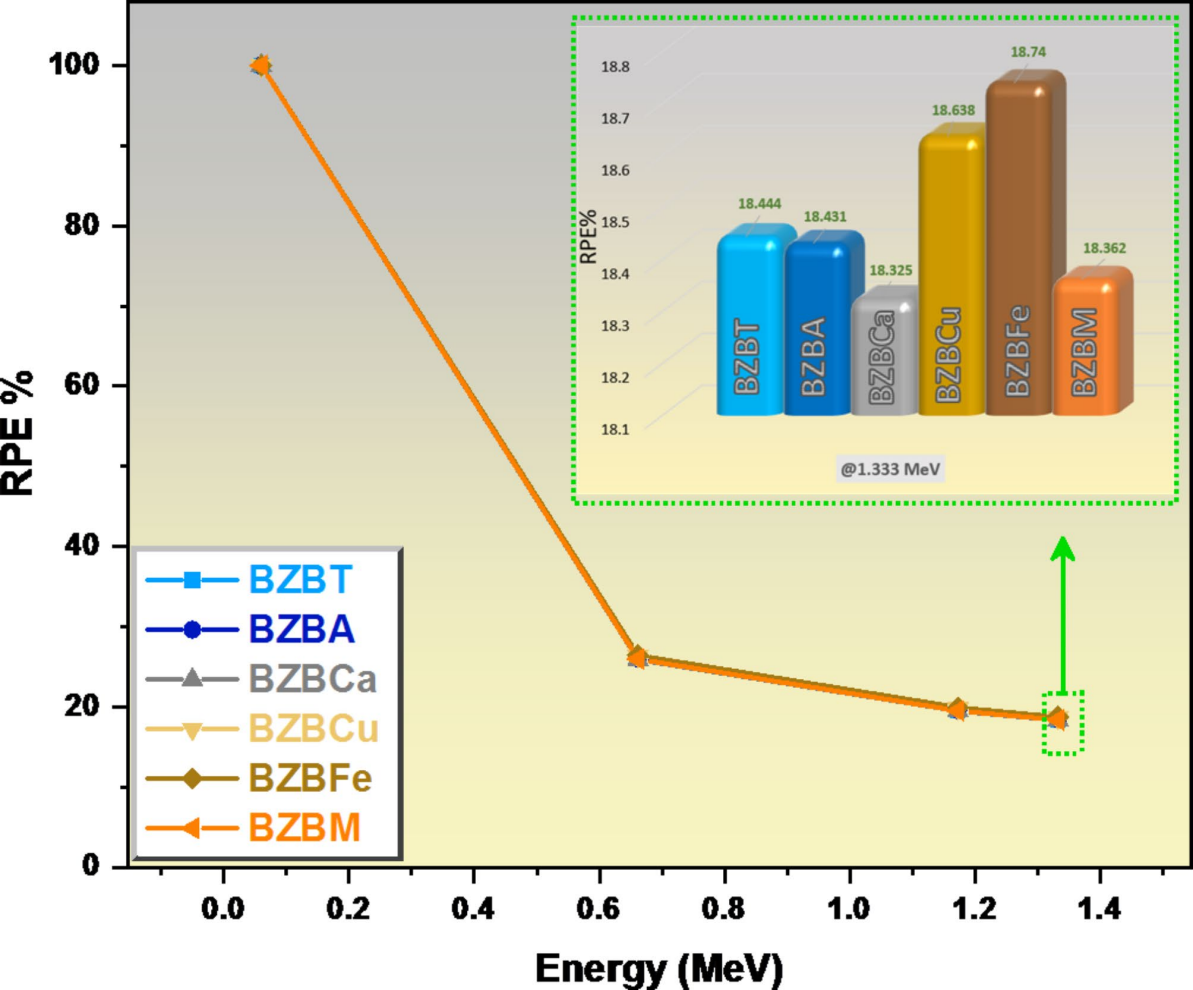
A graph of the radiation protection efficiency against energy E at a thickness of 1.0 cm.

In Fig. 12, we plotted the RPE for the prepared glasses. As expected, all the glass samples under study are 100% efficient at the lowest energy value of 0.060 MeV as shown in Fig. 12. As the energy increases to 0.662 MeV, the RPE dropped suddenly, sample BZBM dropped to 25.98%, sample BZBCa dropped to 25.92%, sample BZBA dropped to 26.03%, sample BZBT dropped to 26.07%, sample BZBCu dropped to 26.34%, while sample BZBFe dropped to 26.43%. We observed further drops in the efficiency of the glasses as we increased the energy of the radiation from 0.662 MeV to 1.333 MeV; sample BZBM dropped to 18.36%, sample BZBCa dropped to 18.32%, sample BZBA dropped to 18.43%, sample BZBT dropped to 18.44%, sample BZBCu dropped to 18.64%, while sample BZBFe dropped to 18.74%. In all the energy range from 0.662 MeV to 1.333 MeV, sample BZBFe has the highest efficiency of attenuating the radiation as such is the best glass for radiation attenuation among the studied glasses.

**Fig. 13:**
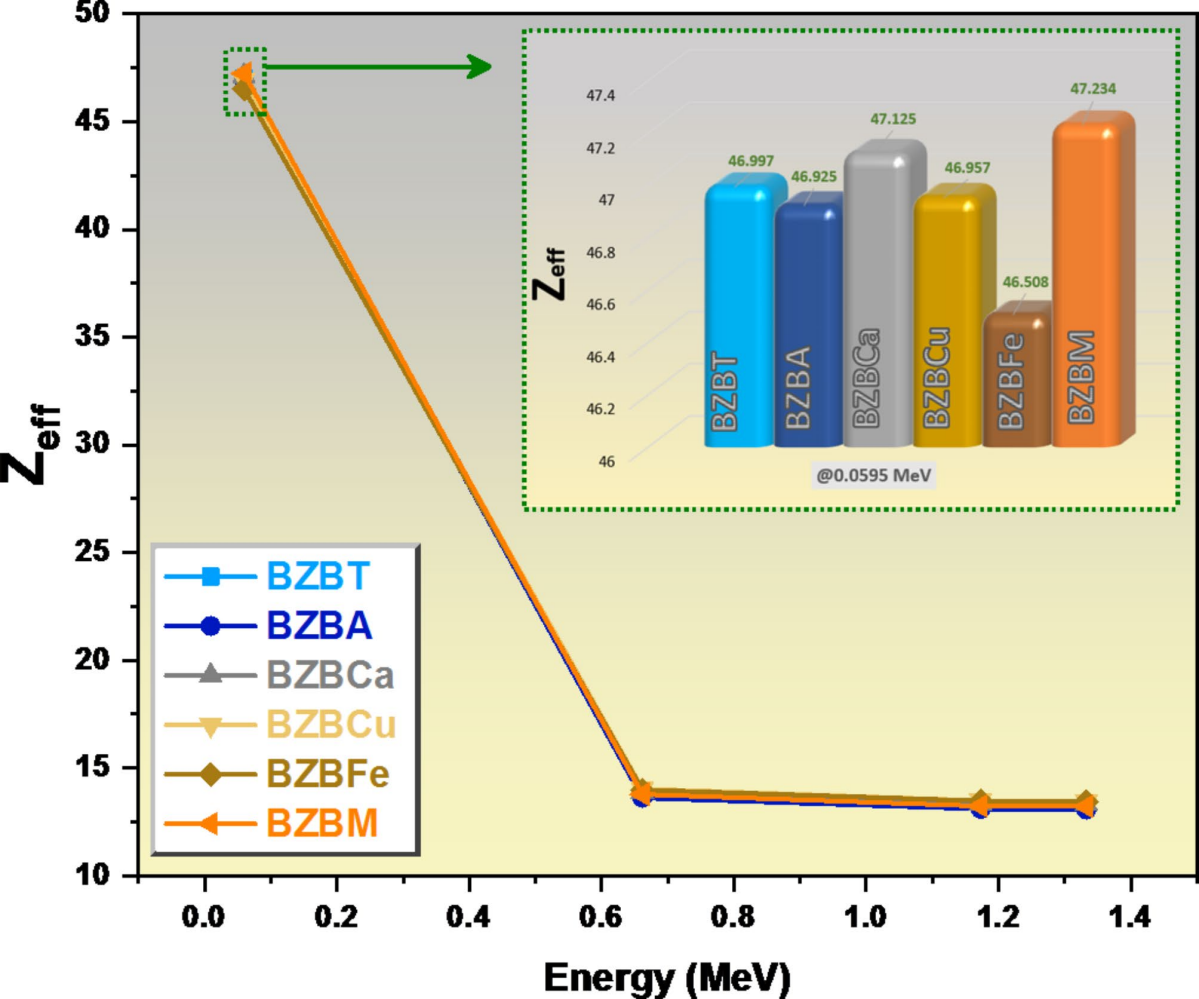
A graph of the Zeff of glass samples against energy E.

The Z_eff_ value was highest for all the glasses at the lowest energy of the radiation as presented in Fig. 13, 0.060 MeV; sample BZBM has a Z_eff_ value of 47.23, sample BZBCa has a Z_eff_ value of 47.13, sample BZBT has a Z_eff_ value of 47.00, sample BZBCu has a Z_eff_ value of 46.96, sample BZBA has a Z_eff_ value of 46.92, while sample BZBFe has a Z_eff_ value of 46.51. As we further increase the radiation’s energy from 0.060 MeV to 0.662 MeV, the Z_eff_ values dropped significantly, sample BZBCu dropped to 14.03, sample BZBFe dropped to 13.99, sample BZBCa dropped to 13.89, sample BZBT dropped to 13.84, sample BZBM dropped to 13.78, while sample BZBA dropped to 13.62. As we increased the energy of the incoming radiation from 0.662 MeV to 1.333 MeV, the dropped in the Z_eff_ values dropped gradually; sample BZBCu dropped to 13.46, sample BZBFe dropped to 13.44, sample BZBCa dropped to 13.33, sample BZBT dropped to 13.28, sample BZBM dropped to 13.21, while sample BZBA dropped to 13.07.

**Fig. 14:**
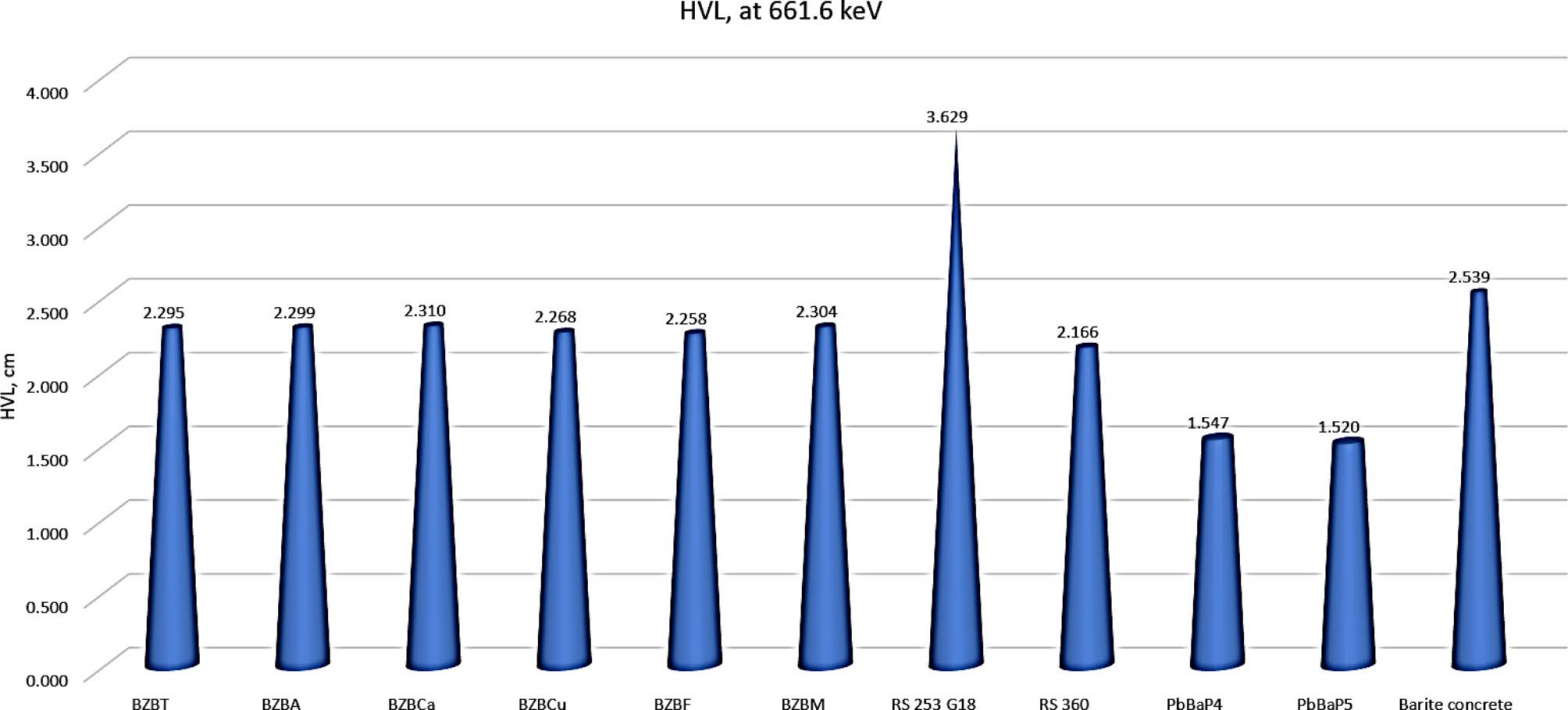
A comparison of glass samples with different commercial glasses and barite concrete.

Finally, the fabricated glass in this work was compared with other commercial glasses such as RS 360 (45SiO_2_–45PbO–10K_2_O + N_2_O)^43^, RS 353 G18 (70SiO_2_–10B_2_O_3_–9K_2_O–8N_2_O–2C_2_O–1BaO)^43^, PbBaP5 (55PbO–5BaO–41P_2_O_5_)^44^ at 661.6 keV. The half value layer parameter was estimated in Fig. 14 as a comparison and the results in the figure indicated that the fabricated glasses can be used as a shielding material against gamma-rays, since it is better the barite concrete^45^ as a shielding material, where the HVL of Barite materials was 2.539 cm at 661.6 keV, while the HVL of fabricated glass ranges from 2.258 up to 2.301 cm.

## Conclusion

The 45B_2_O_3_–20ZnO–30BaO–5X (where X represents CaO, MgO, Al_2_O_3_, CuO and Fe_2_O_3_) glass system’s gamma ray shielding performance was determined by experimental and theoretical techniques. The variation between the two techniques was very small in all discussed gamma energies. The BZBX- glass samples’ LAC was found and it was observed that the sample BZBCu experienced the greatest decrease in LAC value from 16.203 cm^−1^ to 0.306 cm^−1^, while sample BZBA experienced the least LAC decrease from 15.620 cm^−1^ to 0.302 cm^−1^ at 0.0595 MeV. With a further 0.662 to 1.33 MeV energy increase, the decrease in the LAC values became slower with sample BZBCu decreasing from 0.306 cm^−1^ to 0.206 cm^−1^, which was the highest observable reduction in LAC value due to the presence of CuO. Also, sample BZBFe which is the second highest observable decreased in the LAC due to the presence of Fe_2_O_3_. In the 0.662–1.333 MeV energy range, sample BZBFe has the highest efficiency of attenuating the radiation, and as such is the best glass for radiation attenuation among the studied glasses.

## Data Availability

The data that support the findings of this study are available from the corresponding author upon reasonable request.
